# Correlation between quantitative DCE-MRI and pathologic complete response in patients with invasive ductal carcinoma undergoing neoadjuvant systemic therapy

**DOI:** 10.1097/MD.0000000000048122

**Published:** 2026-04-03

**Authors:** Xingrui Wang, Xuehong Xiao, Ang Yang, Shuyan Zeng, Wenxi Chen, Yi Chen, Shien Cui, Zhihua Huang, Yumei Zeng, Xiaoxing Huang

**Affiliations:** a Department of Radiology, Zhongshan City People’s Hospital, Zhongshan, Guangdong, China; b Department of Breast Center, Zhongshan City People’s Hospital, Zhongshan, Guangdong, China; c Department of Pathology, Zhongshan City People’s Hospital, Zhongshan, Guangdong, China.

**Keywords:** dynamic contrast-enhanced magnetic resonance imaging, invasive ductal carcinoma, neoadjuvant systemic therapy, pathologic complete response, peritumoural region

## Abstract

This study aimed to determine the associations between pretreatment quantitative dynamic contrast-enhanced magnetic resonance imaging (DCE-MRI) pharmacokinetic (PK) parameters, post-neoadjuvant systemic therapy (NST) MRI features, and pathologic complete response (pCR) in patients with invasive ductal carcinoma (IDC). Twenty-eight consecutive IDC patients who received NST were retrospectively reviewed. All patients underwent MRI at 3 time points: before NST; after 2 cycles of NST; and before surgery. Continuous and categorical variables were compared between pCR and non-pCR groups using the Mann–Whitney *U* test and Fisher exact test, respectively, with HER2 status adjusted in PK parameter analyses. Partial correlation assessed associations between MRI features and pCR. Select PK parameters were further evaluated using Firth’s penalized-likelihood regression, controlling for covariates. Receiver operating characteristic (ROC) analysis was performed to evaluate predictive performance. Significant differences were detected between groups regarding HER2-positive, luminal B subtype, Miller–Payne, and postoperative lymph node metastasis (*P *< .05). Pretreatment peritumoural extravascular extracellular volume (*V*_e_), post-NST shrinkage pattern, and residual disease were significantly different between the groups (*P *< .05). Partial correlation analysis indicated a positive association between the peritumoural flux rate constant (*K*_ep_) and pCR (*P *< .05). Regression analysis identified the peritumoural *K*_ep_ as a factor influencing the pCR with an area under the curve of 0.756 (95% CI = 0.564–0.947). Our preliminary findings suggested an association between the pCR and pretreatment peritumoural PK parameters, highlighting the potential value of the peritumoural region. These results require further validation in larger prospective studies.

## 1. Introduction

Breast cancer is a leading health concern among women due to the high morbidity and mortality rates. Despite a declining mortality rate, the incidence of breast cancer continues to increase with 20% to 30% of early-stage patients in China progressing to advanced disease.^[1,2]^ Invasive ductal carcinoma (IDC) is the most common pathologic type of breast cancer. Neoadjuvant systemic therapy (NST) facilitates tumor downstaging and conversion of inoperable or non-breast-conserving cases to operable and breast-conserving candidates, thereby enhancing surgical outcomes, reducing recurrence risk, and ultimately improving patient prognosis. The pathologic complete response (pCR) following NST is clinically significant and serves as a guide for subsequent adjuvant therapy.^[3]^

Achieving a pCR after NST is linked to improved disease-free and overall survival.^[4]^ Early prediction of a poor response to treatment is crucial for timely adjustment of treatment programmes and mitigating the increased risk of tumor recurrence due to inadequate treatment.^[5]^ Predicting the pCR prior to surgery could offer valuable insights for tailoring optimal treatment strategies at an earlier stage. However, effective imaging methods for this purpose are lacking.^[6]^

Dynamic contrast-enhanced magnetic resonance imaging (DCE-MRI) is a well-established modality for the preoperative evaluation of breast cancer with high sensitivity affording detailed characterization of tumor morphology, kinetics, and perfusion parameters. These characteristics are critical for predicting the pCR following NST.^[7]^ Indeed, it has been shown that early changes in the transfer constant (*K*^trans^) after 2 NST cycles can predict the ultimate treatment response,^[8]^ while a recent meta-analysis has further confirmed the efficacy of DCE-MRI in dynamically monitoring treatment and the predictive value for a pCR.^[9]^ Consequently, a detailed investigation into the correlation between the pCR and quantitative DCE-MRI-derived tumor characteristics in IDC patients assessed both before and after NST is warranted.

The current study performed breast DCE-MRI and pathologic analyses on IDC patients. We hypothesized the following: there are differences in clinical, pathologic, and MRI features between the pCR and non-pCR groups; the pCR is associated with the clinical, pathologic, and MRI features that exhibit differences; and the associated features are independent predictors of the pCR.

## 2. Materials and methods

### 2.1. Study population

This was a retrospective analysis. This study was approved by the Institutional Review Board of Zhongshan City People’s Hospital (K2022-119) and informed consent was waived by the Institutional Review Board due to the retrospective nature of the study. We retrospectively collected data from consecutive patients with pathologically confirmed IDC between April 2021 and December 2022. The study inclusion criteria were as follows: pathologically confirmed IDC and completion of 3 contrast-enhanced MRI examinations (before treatment, after 2 cycles of NST, and before surgery) with an additional quantitative DCE-MRI scan performed before treatment initiation. The exclusion criteria were as follows: not receiving or not completing NST; not undergoing radical surgery after NST; and poor image quality unsuitable for diagnostic evaluation. The collected information included clinical baseline characteristics, pathologic findings, and MRI examinations, such as age, menopausal status, clinical T and N stage (cT and cN, respectively), pathologic T and N stage (pT and pN, respectively), tumor grade, immunohistochemistry results, Miller–Payne (MP) grade, and postoperative lymph node metastasis (LNM).

The NST regimen was administered in accordance with current clinical guidelines and the principle of individualized medicine. All patients received NST with regimens tailored to the molecular subtypes. The standardized docetaxel, carboplatin, trastuzumab, and pertuzumab (TCbHP) regimen was used for patients with HER2-positive breast cancer. Trastuzumab was administered at a loading dose of 8 mg/kg and a maintenance dose of 6 mg/kg. Pertuzumab was given at a loading dose of 840 mg and a maintenance dose of 420 mg; both were administered every 3 weeks. Both targeted drugs were initiated simultaneously with chemotherapy and a total of 6 cycles of treatment were completed during the perioperative period. Patients with triple-negative breast cancer (TNBC) primarily received sequential anthracycline- and taxane-based therapy combined with a platinum-containing regimen. Some patients received a 4-cycle treatment with high-dose doxorubicin combined with cyclophosphamide, followed by treatment with docetaxel for 4 to 12 weeks, while other patients chose the TEC regimen (docetaxel [70–75 mg/m], epirubicin [75–90 mg/m], and cyclophosphamide [500–750 mg/m]). The docetaxel, doxorubicin, and cyclophosphamide (TAC) regimen was administered every 3 weeks for patients with luminal A or B subtypes.

### 2.2. MRI acquisition

Dynamic contrast-enhanced magnetic resonance imaging was performed using a 3.0-T scanner (Achieva TX; Philips, The Netherlands) with a 4-channel breast coil for data acquisition. Patients were placed in the prone position for imaging with the breasts naturally suspended within the breast coil and the arms placed on either side of the head. Horizontal axis fat suppression, fast-spin echo sequence, *T*1- and *T*2-weighted images, and multiphase dynamic enhanced DCE scans were performed separately.

A fast-spin echo sequence was used in the axial plane with a slice thickness of 3 to 4 mm, a repetition time (TR) of 400 to 600 milliseconds, and an echo time (TE) of 10 to 20 milliseconds for *T*1-weighted images. A fast-spin echo sequence was also used in the axial plane with a slice thickness of 3 to 4 mm, a TR of 3000 to 4000 milliseconds, and a TE of 80 to 100 milliseconds for *T*2-weighted images. Horizontal axis fat suppression was performed in the axial plane with a slice thickness of 3 to 4 mm, a TR of 400 to 600 milliseconds, and a TE of 10 to 20 milliseconds.

The DCE-MRI protocol used a 3D *T*1-weighted axial sequence. Baseline *T*1 values (*T*1_0_) were obtained from pre-contrast scans using 2 separate acquisitions with flip angles of 5° and 15°, respectively, while keeping all other sequence parameters identical. The acquisition times for these scans were 58 seconds and 52 seconds, respectively. Corrections for B1 field inhomogeneity were applied to ensure accurate *T*1 quantification. After 1 minute of dynamic enhanced scanning, gadopentetic acid dimeglumine salt (Gd-DTPA; Magnevist, Bayer AG, Leverkusen, Germany) was injected as an intravenous bolus (0.1 mmol/kg) using an MR-compatible power injector (Spectris; Bayer HealthCare, Leverkusen, Germany) at a flow rate of 2.0 mL/s, followed by a 20-mL saline flush. Continuous scanning was performed 25 times with a single and total time of 15.5 seconds and 7 minutes 4 seconds, respectively. DCE parameters were as follows: TR = shortest; TE = shortest; flip angle = 10°; number of signal average (NSA) = 1; layer thickness = 4 mm; layer thickness = 0 mm; and matrix = 340 × 340. During the dynamic contrast-enhanced acquisition, the signal intensity at each time point was converted into the corresponding longitudinal relaxation time, *T*1(*t*), by applying an inverse solution of the sequence-specific signal model. The contrast agent concentration, *C*(*t*), was then calculated using the following formula:


$$\begin{array}{l} \;C\left(t\right)=\frac{1}{r_{1}}\left(\frac{1}{T1\left(t\right)}-\frac{1}{T1_{0}}\right), \end{array}$$

where *C*(*t*) is the tissue concentration of the contrast agent at time point *t*, *T*1_0_ is the baseline longitudinal relaxation time measured prior to contrast agent injection, *T*1(*t*) is the longitudinal relaxation time at post-contrast time point *t*, and *r*_1_ is the longitudinal relaxivity constant of the specific gadolinium chelate used at the scanner’s field strength.

### 2.3. Data analysis

#### 2.3.1. Image post-processing

Dynamic contrast-enhanced magnetic resonance imaging images were independently analyzed by 2 radiologists specializing in breast imaging, with 5 and 10 years of experience, respectively. Conventional techniques were used, such as fixed pads and respiratory gating, to minimize patient movement to the greatest extent during the scanning process. A strict multistep registration and quality control process was implemented to ensure the spatial consistency of the dynamic sequences. All dynamic images underwent preliminary inter-frame alignment checks in the post-processing workstation. Specialized image analysis software was used to perform visual verification of the images, especially on the key layers where the target lesion was located, on a frame-by-frame and layer-by-layer basis. This process aimed to eliminate the potential impact of movement on the quantitative analysis of the pharmacokinetic (PK) curves. The data were regarded as unqualified and excluded in cases with motion artifacts.

Following image preprocessing and rigorous motion correction of key imaging planes, individualized arterial input function (AIF) was obtained by manually delineating the thoracic aorta on a Philips workstation using dedicated quantitative analysis software. Subsequently, a retrospective PK analysis was performed based on the extended Tofts model (ETM). The formula is as follows:


$$\begin{array}{l} C\left(t\right)=v_{p}C_{a}\left(t\right)+K^{trans}e^{-tk_{ep}}*C_{a}\left(t\right), \end{array}$$

where *C*(*t*) is the contrast concentration in tissue, *V*_p_ is the fractional plasma volume (also referred to as the volume fraction of the plasma space), *C*_a_(*t*) is the AIF, the contrast concentration in the feeding artery (see explanation in the section below), *K*^trans^ is the transfer constant between blood plasma and the extravascular extracellular space (EES), and *K*_ep_ is the rate constant between the EES and blood plasma.

This process yielded the following quantitative parameters: volume transfer constant (*K*^trans^); rate constant (*K*_ep_); extravascular extracellular volume fraction (*V*ₑ); and plasma volume fraction (*V*_p_). Among these parameters, *V*_e_ was derived from the ratio, *K*^trans^:*K*_ep_).

**Figure 1.**
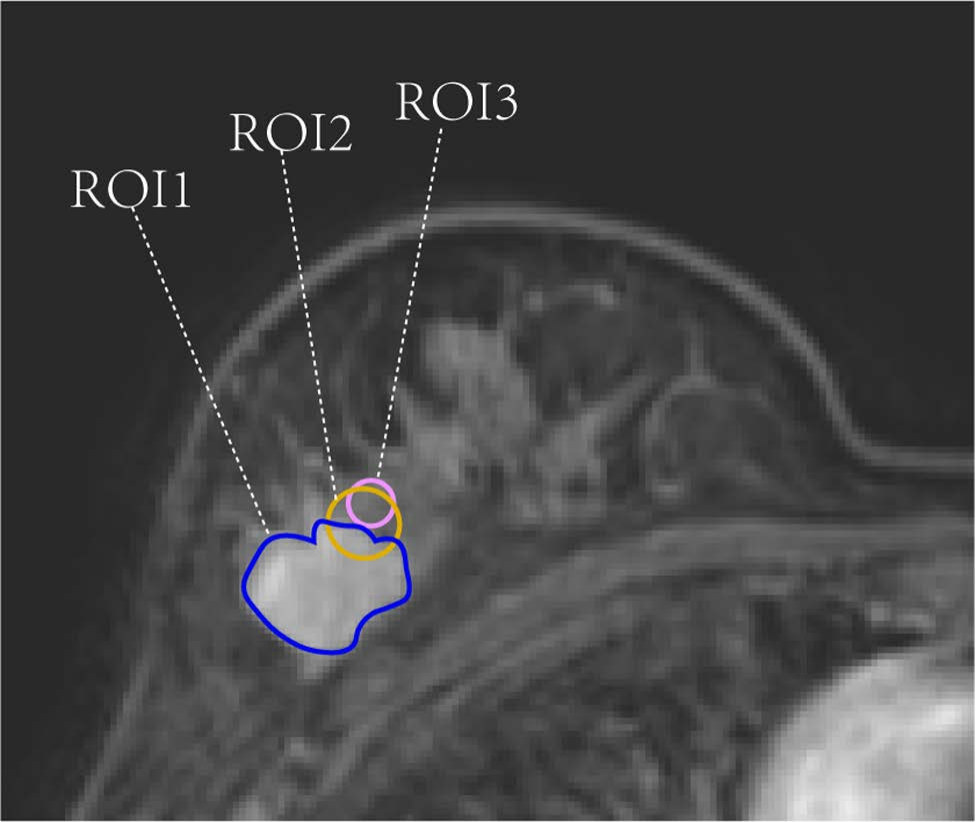
Schematic representation of region‑of‑interest (ROI) delineation. ROI = region of interest.

Region of interest (ROI) analysis was performed by delineating 3 ROIs on the central tumor slice and the 2 adjacent slices (superior and inferior), resulting in a total of 9 ROIs. The average values from these ROIs were used as the final data for each of the 3 defined regions. The specific ROIs were delineated as follows: ROI1 was delineated as an amorphous region encompassing the entire lesion. The center was defined as the intersection point of the longest diameter of the tumor and the perpendicular line on that imaging slice. ROI2 was defined as a roughly circular region with an area of 50 mm^2^, representing the tumor-normal parenchyma interface. The center was placed at the intersection of the tumor border and the line connecting the center of the ROI1 to the nipple. ROI3 was defined as a roughly circular peritumoral region (area = 30 mm^2^) located within 9 mm^[10,11]^ outside the tumor border. The center was placed on the line connecting the center of ROI1 to the nipple with the perimeter touching the center point of ROI2. For a schematic illustration, see Figure 1.

The time-intensity curve (TIC) type for the tumor was determined based on the data obtained from ROI1.

#### 2.3.2. Image analysis

Baseline assessments were performed using pretreatment MRI. These assessments included an evaluation of the background parenchymal enhancement (BPE) and characterization of the tumor morphologic features: location; number; type; size; margin; enhancement pattern; and TIC.

Tumor response was assessed at 2 specific time points. The tumor shrinkage pattern was evaluated on MRI images acquired after 2 cycles of NST. Subsequently, the extent of residual disease (RD) was evaluated on MRI images obtained after completion of NST.

Background parenchymal enhancement was classified into 4 types according to the 2016 American College of Radiology Breast Imaging Reporting and Data System, with the following characteristics: A) almost no enhancement; B) mild background enhancement; C) moderate background enhancement; and D) severe background enhancement. The size was recorded as the maximum diameter in 3 dimensions. Tumor type was categorized as enhanced or non-enhanced mass groups with clear or indistinct margins and the number of lesions classified as single or multiple. The enhancement pattern was classified as homogeneous or heterogeneous.

Tumor shrinkage pattern was categorized into the following 3 types^[12,13]^: type I (complete response), indicating favorable treatment efficacy; type II (concentric shrinkage [CS]), defined by a decrease in the longest tumor diameter posttreatment with or without residual foci around the mass, suggesting effective treatment; and type III (non-CS), encompassing any pattern other than CS, including fragmented or multinodular lesions, stable disease, or tumor progression, indicating poor treatment efficacy.

Residual disease was defined as the assessment of remaining tumor status based on the preoperative MRI performed after completion of NST.

#### 2.3.3. Pathologic analysis

Postoperative pathology and immunohistochemistry results from pathologic sections were analyzed by a professional pathologist, determining the breast cancer molecular subtypes, lymph node metastasis, and Miller–Payne (MP) scores. Breast cancer was classified into 4 subtypes based on marker expression according to the breast cancer clinical treatment guidelines (2022): The luminal A subtype is estrogen receptor (ER)-positive, progesterone receptor (PR) positivity ≥ 20%, HER2-negative, and Ki-67 index ≤ 14%. The luminal B subtype is ER- and/or PR-positive, HER2-negative with a Ki-67 index > 14% or PR expression ≤ 20%. The HER2-positive subtype has HER2 overexpression with an immunohistochemistry (IHC) score of 3+ or an IHC score of 2+ with confirmation of gene amplification by fluorescence in situ hybridization (FISH). The TNBC subtype is negative for ER, PR, and HER2 expression.

The levels of ER and PR expression were stratified as high (>10%) or low (≤10%) and Ki-67 expression was similarly dichotomized into high (>20%) or low (≤20%) groups. HER2 status and LNM were classified as positive or negative.

Miller–Payne was classified into 5 grades according to the MP scoring system: Grade 1 (G1) is infiltrating cancer cells that show no changes or only a few changes with the overall number of cancer cells remaining unchanged. Grade 2 (G2) is infiltrating cancer cells that are slightly reduced in number but the total number remains high with a ≤30% reduction. The grade 3 (G3) infiltrating cancer cell number ranges from 30% to 90%. Grade 4 (G4) is infiltrating cancer cells significantly decreased in number > 90% with only scattered small clusters or individual cancer cells remaining. Grade 5 (G5) is characterized by no infiltrating cancer cells present in the tumor bed, although in situ ductal carcinoma may exist.

According to the MP system, good responders (grades 4–5) and poor responders (grades 1–3) were defined with only grade 5 representing a pCR; all other grades (1–4) constituted non-pCR.

Pathologic complete response was defined as the absence of invasive cancer in the breast and axillary lymph nodes after NST, corresponding to ypT0/Tis and ypN0.

## 3. Statistical analysis

Continuous variables were tested for normality using the Shapiro–Wilk test. Normally distributed data are presented as the mean ± standard deviation, while non-normally distributed data are presented as the median (IQR). Categorical variables are expressed as numbers (percentages). Group comparisons between the pCR and non-pCR groups were performed using the Mann–Whitney *U* test for continuous variables and the Fisher exact test for categorical variables. HER2 status was included as a covariate in the PK parameter comparisons of MRI features. Partial correlation analysis was used to assess the relationship between MRI features and pCR with HER2 status, luminal B subtype, and postoperative LNM as covariates. The MRI features identified through this screening process were used in subsequent analyses. Spearman correlation analysis was used to assess the associations between post-NST MRI features and pathologic outcomes. Firth penalized likelihood regression was used to analyze the univariate association of the selected features with pCR and sequential adjustment for the abovementioned covariates. In addition, receiver operating characteristic (ROC) curves were plotted and the area under the curve (AUC), sensitivity, and specificity were calculated. For quantitative measurements, such as tumor size and PK parameters, interobserver agreement was evaluated using the intraclass correlation coefficient (ICC). All statistical analyses were performed using SPSS (version 27; IBM SPSS Statistics for Windows, Armonk), R (version 4.5.2; R Foundation for Statistical Computing, Vienna, Austria), and GraphPad Prism (version 10.3.1; GraphPad Software, Boston). A *P*-value < .05 was considered statistically significant.

## 4. Results

### 4.1. Sample size and statistical power

The study included 29 participants. An a priori sample size calculation (G*Power 3.1; α = 0.05, 2-tailed; power = 0.72; effect size |ρ| = 1.1) showed that 28 samples were needed to detect a moderate effect.

### 4.2. Clinical characteristics

**Figure 2.**
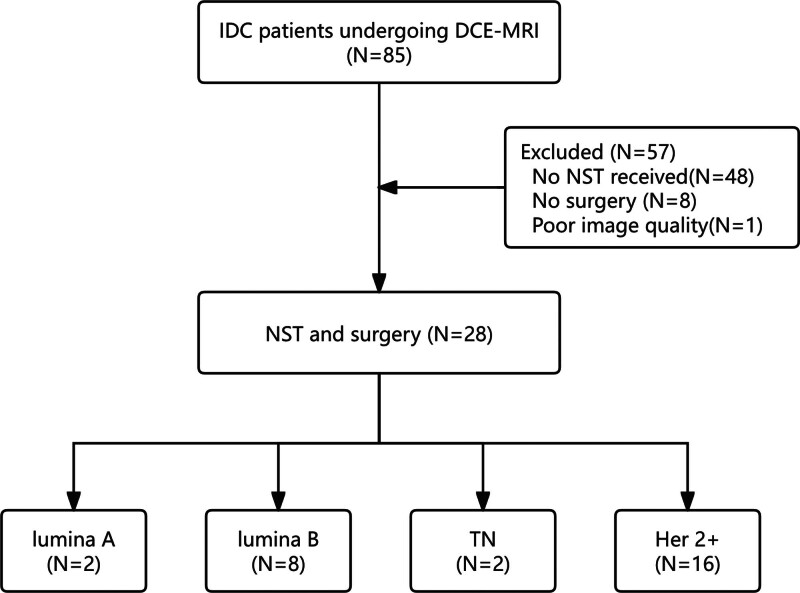
Flowchart of patient enrollment and study assessment. IDC = invasive ductal carcinoma, DCE-MRI = dynamic contrast-enhanced magnetic resonance imaging, NST = neoadjuvant systemic therapy, TN = triple-negative.

A total of 28 patients (29 tumors [1 patient had bilateral breast cancer]) were included in this study. The median age was 50 years (IQR, 45–57). The enrollment process is detailed in Figure 2. Comparative analysis revealed that HER2-positive (*P* = .003), luminal B subtype (*P* = .033), MP (*P* < .001), and postoperative LNM (*P* < .001) differed significantly between the pCR and non-pCR groups, although no significant differences were detected in menopausal status (*P* = 1.000), clinical T stage (*P* = .361), N stage (*P* = .260), grade (*P* = .119), luminal A (*P* = .557), triple-negative status (*P* = .557), or other protein markers (Table 1).

**Table 1:** Comparison of clinical and pathologic characteristics between the pCR and non-pCR groups.

Characteristic	Pathologic response			
	pCR (n = 9)	Non-pCR (n = 20)	Fisher	*P* value
Age, median (IQR)	50 (49, 57)	49 (43, 57)		.432
Menopausal status, n (%)			0.001	1.000
Premenopausal	5 (56)	11 (55)		
Postmenopausal	4 (44)	9 (45)		
Clinical tumor stage, n (%)			3.387	.361
T2	6 (67)	18 (90)		
T3 or T4	3 (33)	2 (10)		
Clinical nodal stage, n (%)			5.362	.260
N0	1 (10)	4 (21)		
N1, N2, or N3	9 (90)	15 (79)		
Grade, n (%)			5.323	.119
II	3 (33)	13 (65)		
III or IV	6 (67)	7 (35)		
ER, n (%)			0.606	.688
≤10%	5 (56)	8 (40)		
>10%	4 (44)	12 (60)		
PR, n (%)			1.436	.412
≤10%	7 (78)	11 (55)		
>10%	2 (22)	9 (45)		
Ki67, n (%)			1.436	.412
≤20%	2 (22)	9 (45)		
>20%	7 (78)	11 (55)		
HER2-positive, n (%)	9 (100)	8 (40)	12.416	.003**
Luminal A, n (%)	0 (0)	2 (10)	1.552	.557
Luminal B, n (%)	0 (0)	8 (40)	7.242	.033*
TNBC, n (%)	0 (0)	2 (10)	1.552	.557
MP, n (%)			29.422	.001**
Good responders (G5 or G4)	9 (100)	3 (15)		
Poor responders (G1–G3)	0 (0)	17 (85)		
Postoperative LNM, n (%)			17.675	.001**
Yes	5 (56)	15 (75)		
No	4 (44)	5 (25)		

ER = estrogen receptor, HER2-positive = human epidermal growth factor receptor 2 overexpression, IQR = interquartile range, LNM = lymph node metastasis, MP = Miller–Payne, pCR = pathologic complete response, PR = progesterone receptor, TNBC = triple-negative breast cancer.

* *P* < .05.

** *P* < .01.

### 4.3. MRI features in pCR

**Figure 3.**
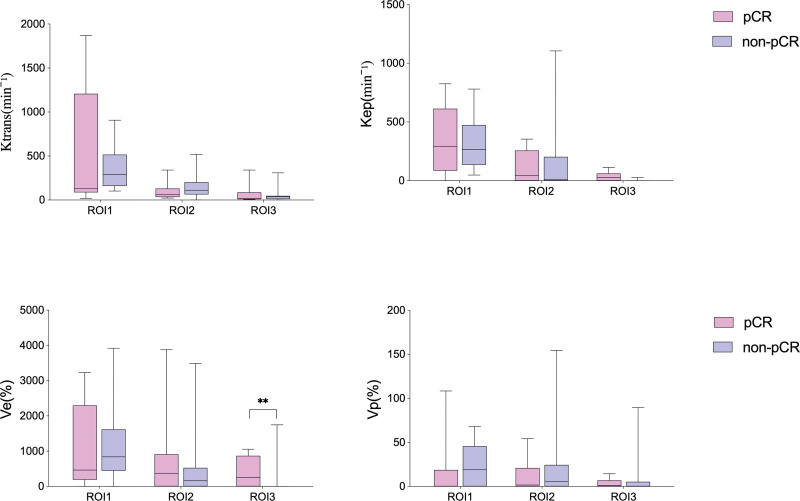
Comparisons of PK parameters between pCR and non-pCR group, adjusted for HER2. *K*^trans^ = transfer constant, *K*_ep_ = flux rate constant, pCR = pathologic complete response, PK = pharmacokinetic, ROI = region of interest; *V*_e_ = extravascular extracellular volume, *V*_p_ = capillary plasma volume.

Among the pretreatment MRI features, only peritumoral *V*_e_ from the PK parameters demonstrated a significant difference between the pCR and non-pCR groups after adjusting for the covariate HER2 status (*P* = .008). None of the other pretreatment MRI features showed significant intergroup differences (Fig. 3).

The tumor shrinkage pattern and RD showed significant differences between the response groups for the post-NST MRI assessment (*P* = .034 and *P* = .01, respectively). Detailed results are presented in Tables 2 and 3.

**Table 2:** Comparison of MRI features between groups.

Characteristic	Pathologic response			
	pCR (n = 9)	Non-pCR (n = 20)	Fisher	*P* value
Size, median (IQR)	45.6 (29.9,57.7)	34 (28.4,44.0)		.187
Location, n (%)			0.353	.694
Right	6 (67)	11 (55)		
Left	3 (33)	9 (45)		
BPE, n (%)			3.819	.401
A or B	5 (56)	16 (80)		
C or D	4 (44)	4 (20)		
Type, n (%)			0.908	.431
Mass	7 (78)	12 (60)		
Non-mass	2 (22)	8 (40)		
Margin, n (%)			0.233	.694
Clear	4 (44)	7 (35)		
Unclear	5 (56)	13 (65)		
Number of lesions, n (%)			1.696	.237
Single	5 (56)	6 (30)		
Multiple	4 (44)	14 (70)		
Enhancement pattern, n (%)			0.731	.568
Homogeneous	2 (22)	2 (10)		
Heterogeneous	7 (78)	18 (90)		
TIC, n (%)			3.130	.345
I	2 (22)	1 (5)		
II	7 (78)	17 (85)		
III	0 (0)	2 (10)		
Shrinkage pattern, n (%)			5.946	.034*
I	5 (56)	3 (15)		
II	4 (44)	11 (55)		
III	0 (0)	6 (30)		
RD, n (%)			8.945	.010**
Yes	2 (22)	16 (80)		
No	7 (78)	4 (20)		

BPE = background parenchymal enhancement, IQR = interquartile range, MRI = magnetic resonance imaging, pCR = pathologic complete response, RD = residual disease, TIC = time-signal intensity curve.

* *P* < .05.

** *P* < .01.

**Table 3:** Intergroup comparison of PK parameters (pCR vs non-pCR), adjusted for HER2.

	pCR	Non-pCR	*W* statistic	*P* value
Tumor region				
*K* ^trans^	129.16 (99.98–918.1)	293.04 (162.477–443.657)	105	.4943
*K*_ep_	290.34 (170.29–463.79)	265.17 (152.203–410.488)	96	.7954
*V*_e_	465.05 (379.69–2142.16)	931.215 (532.17–1774.22)	106	.4649
*V*_p_	0.31 (0–9.59)	18.925 (0–45.448)	100	.6527
Interface region				
*K* ^trans^	61.44 (34.78–126.63)	110.27 (65.1–193.158)	117	.2116
*K*_ep_	43.93 (0–198.87)	12.45 (0–196.142)	114	.2638
*V*_e_	374.01 (0–738.07)	160.325 (0–489.123)	86	.8678
*V*_p_	1.5 (0–15.92)	5.43 (0–21.295)	125	.1006
Peritumoral region				
*K* ^trans^	16.54 (9.71–85.38)	37.32 (14.998–46.627)	99	.6886
*K*_ep_	28.08 (0–37.42)	0 (0–0)	63	.1892
*V*_e_	251.38 (0–754.58)	0 (0–0)	36	.008*
*V*_p_	1.74 (0.07–7.05)	0 (0–3.265)	122	.1321

*K*^trans^ = transfer constant, *K*_ep_ = flux rate constant, pCR = pathologic complete response, PK = pharmacokinetic, *V*_e_ = extravascular extracellular volume, *V*_p_ = capillary plasma volume.

* *P* < .01.

The ICC for interobserver consistency among size, ROI1, ROI2, and ROI3 were 0.978 (0.913–0.0992), 0.955 (0.935–0.969), 0.942 (0.916–0.959), 0.994 (0.992–0.996), respectively.

### 4.4. Correlation analysis

After adjusting for covariates (HER2 status, luminal B subtype, and postoperative LNM), peritumoral *K*_ep_ remained positively correlated with the pCR (partial *r* = 0.450, *P* = .021; Table 4). None of the other MRI features correlated significantly with the pCR.

**Table 4:** Partial correlations between PK parameters and pCR.

	Mean	SD	Partial *r*	*P* value
Tumor region				
*K* ^trans^	425.209	428.795	0.2519	.2145
*K*_ep_	327.847	239.04	−0.0747	.717
*V*_e_	1451.067	1674.469	−0.2208	.2783
*V*_p_	21.543	28.05	−0.0244	.9057
Interface region				
*K* ^trans^	129.931	126.988	−0.3706	.0624
*K*_ep_	133.787	230.785	−0.3739	.0599
*V*_e_	563.963	972.983	0.1631	.4258
*V*_p_	17.323	31.933	−0.1084	.5981
Peritumoral region				
*K* ^trans^	59.277	83.058	0.0552	.7888
*K*_ep_	11.452	27.129	0.4502	.021*
*V*_e_	552.591	1987.133	0.0711	.7299
*V*_p_	26.892	110.553	0.1993	.3291

*K*^trans^ = transfer constant, *K*_ep_ = flux rate constant, pCR = pathologic complete response, PK = pharmacokinetic, *V*_e_ = extravascular extracellular volume, *V*_p_ = capillary plasma volume.

* *P* < .05.

Post-NST MRI features, the tumor shrinkage pattern, and RD were significantly correlated with molecular subtypes. Specifically, the shrinkage pattern showed a negative correlation with HER2-positive breast cancer (*r* = −0.591, *P* < .001) and a positive correlation with the luminal B subtype (*r* = 0.399, *P* = .032). Similarly, RD was negatively correlated with HER2-positive breast cancer (*r* = −0.657, *P* < .001) and positively correlated with the luminal B subtype (*r* = 0.482, *P* = .008).

### 4.5. Univariate analysis using Firth penalized likelihood regression

**Figure 4.**
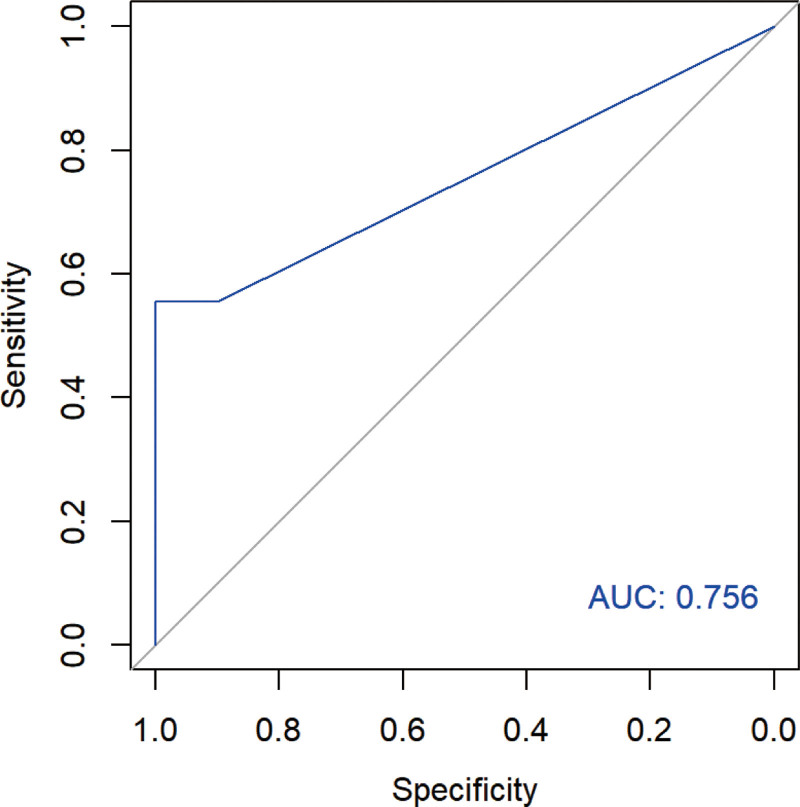
ROC curve of peritumoral *K*_ep_ for predicting pCR. AUC = area under the curve, *K*_ep_ = flux rate constant, pCR = pathologic complete response, ROC = receiver operating characteristic.

Firth logistic regression analysis revealed peritumoral *K*_ep_ as a significant predictor of pCR when adjusting for HER2 status alone (OR = 1.075, 95% CI = 1.003–1.290; *P *= .032) and when adjusting for HER2 status and luminal B subtype (OR = 1.069, 95% CI = 1.003–1.271; *P* = .034). However, this association was no longer significant after further adjustment for postoperative LNM (OR = 1.045, 95% CI = 0.995–1.250; *P *= .110). For predicting pCR, peritumoral *K*_ep_ achieved an AUC of 0.756 (95% CI = 0.564–0.947), with a sensitivity of 0.556 (95% CI = 0.200–0.875), a specificity of 1.000, an accuracy of 0.862, a positive predictive value of 1.000, and a negative predictive value of 0.833; the details are provided in Figure 4.

In contrast, peritumoral *V*_e_ had no significant association with the pCR across all adjusted models: When controlling for HER2 status (OR = 1.000, 95% CI = 1.000–1.024, *P *= .224), for HER2 and luminal B subtype (OR = 1.000, 95% CI = 1.000–1.022, *P *= .296), and for HER2 status, luminal B subtype, and postoperative LNM (OR = 1.000, 95% CI = 1.000–1.011; *P *= .337).

**Figure 5.**
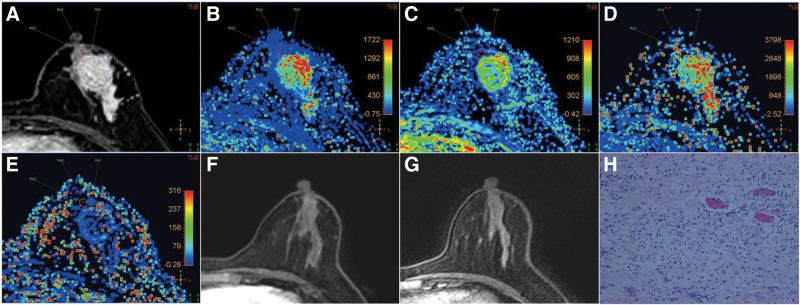
MRI images before and after treatment in a 50-year-old woman with grade II invasive ductal carcinoma. (A) Axial pretreatment DCE-MRI. The image shows the placement of 3 regions of interest (ROIs): ROI1: whole tumor area; ROI2: tumor-normal parenchyma interface; ROI3: peritumoral region. (B–E) Pharmacokinetic (PK) parameter maps. Maps for *K*^trans^, *K*_ep_, *V*_e_, and *V*_p_ are displayed. Peritumoral *V*_e_ demonstrated a significant difference between pCR groups and peritumoral *K*_ep_ was correlated with pCR (^*^P < .05, ^**^P < .01). (F–G) Post-NST axial contrast-enhanced MRI. The tumor exhibited a type I shrinkage pattern after NST. Posttreatment MRI revealed no residual tumor. (H) Surgical pathologic specimen. Pathologic assessment confirmed a Miller–Payne grade of G5, indicating achievement of a pathologic complete response (pCR). DCE-MRI = dynamic contrast-enhanced magnetic resonance imaging, *K*^trans^ = transfer constant, *K*_ep_ = flux rate constant, MRI = magnetic resonance imaging, NST = neoadjuvant systemic therapy, pCR = pathologic complete response, PK = pharmacokinetic, ROI = region of interest, *V*_e_ = extravascular extracellular volume, *V*_p_ = capillary plasma volume.

A typical case involving a patient diagnosed with left breast IDC is presented in Figure 5, which shows MRI images before and after treatment in a 50‑year‑old woman with grade II IDC.

## 5. Discussion

The current study preliminarily investigated the association between pretreatment multiregional DCE-MRI PK parameters and a pCR. An important finding was that pretreatment peritumoral *K*_ep_ and *V*_e_ were associated with a pCR and peritumoral *K*_ep_ in particular showed potential as a factor influencing the pCR after NST. However, after adjusting for all covariates, this association was no longer statistically significant. Nevertheless, the peritumoral region warrants further investigation.

It has been established that the peritumoral area often contains biologically relevant information, such as peritumoral lymphatic vessel invasion (PLVI), lymphocyte infiltration, and edema.^[14]^ Previous studies have indicated that features extracted from 0 to 3 mm peritumoral ring correlate significantly with tumor-infiltrating lymphocyte density and that characteristics from 9 to 12 mm peritumoral region outperform intratumoral features in identifying HER2 status and predicting response to targeted therapy.^[15]^ Peritumoral edema is a characteristic manifestation of invasive breast cancer. Peritumoral edema reflects increased vascular permeability due to the rapid proliferation of immature tumor vasculature, as well as a state of cytokine release in the surrounding tissue.^[16]^ Cellular composition in the peritumoral region, including enrichment of B cells, immature dendritic cells, and neutrophil infiltration, has also been linked to tumor invasiveness.^[17]^ Moreover, peritumoral lipoproteins may promote tumor growth.^[18]^ Multiple studies indicate that the peritumoral microenvironment is closely associated with tumor progression, metastasis, and prognosis.^[19,20]^ Gasparini et al^[21]^ identified peritumoural lymphovascular invasion (PLVI) as an independent predictor of relapse-free survival (RFS) in node-negative primary breast cancer patients. Similarly, peritumoral edema has been significantly associated with RFS in TNBC^[22]^ and posttreatment edema correlates with overall survival (OS).^[23]^

Recent radiomic studies have highlighted the significant role of the peritumoral microenvironment. Peritumoral radiomic features can be used to predict ductal carcinoma in situ, identify tumor HER2 and Ki67 status, guide personalized treatment, and consequently influence patient prognosis.^[14,24]^ Combining intratumoral and peritumoral radiomic features improves the accuracy of pretreatment prediction of a pCR^[14,25,26]^

Therefore, we hypothesize that DCE PK parameters in the peritumoral microenvironment may hold value for predicting a pCR after NST in breast cancer. An elevated peritumoral *K*_ep_ likely reflects greater blood flow and vascular permeability in this region. Subsequent changes in peritumoral *V*_e_ may enhance the delivery of chemotherapeutic agents, thereby contributing to treatment efficacy. These findings are supported by Kettunen et al, who reported that cellular density and interstitial space in the peritumoral area are linked to breast cancer invasion and prognostic indicators.^[11]^ More importantly, these parameters may indirectly signify an immunologically active host-response microenvironment that can cooperate more effectively with chemotherapy to eliminate tumor cells, which is consistent with the observation by Braman et al^[25]^ that a brisk lymphocytic response in the peritumoral area favors a pCR.^[27,28]^ This finding suggests that the peritumoral region may deserve evaluation as a distinct biological entity. However, our findings are preliminary and require validation in larger, prospective cohorts.

The results of this study demonstrated that post-NST MRI features (specifically, the shrinkage pattern and RD) exhibited significant differences between the response groups and were associated with molecular subtypes. The tumor shrinkage pattern reflects multiple tumor characteristics, including gene expression profiles, molecular subtypes, enhancement patterns, and intratumoural microbiota status.^[29–31]^ Different shrinkage patterns may serve as early indicators of NST response and have been linked to specific survival outcomes.^[12,13,32–34]^

Previous studies have shown that CS is a common pattern in HER2-positive breast cancer and TNBCs. Concentric shrinkage has been associated with a more favorable prognosis in luminal-type tumors compared to non-CS patterns,^[12]^ as well as improved disease-free survival (DFS) and OS.^[35]^ Heacock et al^[36]^ similarly observed that tumor shrinkage was correlated with the pCR in HER2-positive patients receiving targeted therapy, which aligns with our findings. Therefore, early MRI assessment of the shrinkage pattern may help to improve patient prognosis, survival, and quality of life.

Prior research has reported a high concordance between MRI-assessed RD and pathologic findings.^[37]^ However, the MRI evaluation of RD can be influenced by molecular subtype and shrinkage pattern, potentially leading to underestimation of residual tumor burden,^[12]^ a result consistent with the present study. These observations require further validation in larger sample cohorts.

### 5.1. Limitation

This study had several limitations. First, the relatively small sample size constrained the statistical power. Therefore, the results should be interpreted with caution and validated in a larger cohort. Accordingly, we used Firth penalized-likelihood regression, which is suitable for small-sample analysis. Second, all pCR events occurred in the HER2-positive subtype, which is consistent with the known efficacy of dual HER2-targeted therapy (trastuzumab plus pertuzumab) in this population. This led to a strong association between HER2 status and the pCR, limiting the generalizability of our findings to other subtypes, such as HER2-negative breast cancer. Although HER2 status was adjusted for as a key covariate in the regression models, residual confounding cannot be fully excluded. Third, this study was designed as an exploratory analysis aimed at screening multiparametric DCE-MRI features. Thus, despite being guided by biological rationale and adjusted for relevant variables, a strict correction for multiple comparisons was not performed. The results should be regarded as hypothesis-generating rather than confirmatory. Fourth, treatment regimens varied inherently across molecular subtypes according to the “treatment-by-subtype” principle (e.g., HER2‑positive patients received dual HER2 blockade). This clinical heterogeneity, while reflecting real‑world practice, makes it statistically challenging to completely disentangle the effect of treatment from the molecular subtype. Finally, although manual ROI delineation showed good inter‑observer agreement, manual ROI delineation may introduce operator‑dependent variability. Future studies would benefit from automated segmentation techniques to improve reproducibility. In summary, the findings of this study are preliminary and require validation in larger, prospective, and treatment‑homogeneous cohorts. Further research is also needed to explore the predictive value of these imaging biomarkers across different molecular subtypes of breast cancer.

## 6. Conclusions

In conclusion, pretreatment peritumoral PK parameters are associated with the pCR in breast cancer and peritumoral *K*_ep_ may be a contributing factor to pCR. In addition, tumor shrinkage patterns after NST have an association with the pCR. However, these key findings should be considered preliminary and warrant further investigation in larger, prospective patient cohorts.

## Author contributions

**Conceptualization:** Xiaoxing Huang.

**Data curation:** Xingrui Wang, Xuehong Xiao, Ang Yang, Shuyan Zeng, Wenxi Chen, Yi Chen, Shien Cui, Zhihua Huang, Yumei Zeng.

**Formal analysis:** Xingrui Wang, Ang Yang, Shien Cui, Xiaoxing Huang.

**Funding acquisition:** Xiaoxing Huang.

**Investigation:** Xingrui Wang, Ang Yang, Shien Cui.

**Methodology:** Xingrui Wang, Ang Yang, Shien Cui, Xiaoxing Huang.

**Project administration:** Xuehong Xiao, Xiaoxing Huang.

**Resources:** Yi Chen, Shien Cui, Zhihua Huang, Yumei Zeng.

**Supervision:** Xuehong Xiao, Xiaoxing Huang.

**Visualization:** Xingrui Wang, Ang Yang, Shien Cui, Xiaoxing Huang.

**Writing – original draft:** Xingrui Wang, Xuehong Xiao, Ang Yang, Shuyan Zeng, Wenxi Chen, Yi Chen, Shien Cui, Zhihua Huang, Yumei Zeng, Xiaoxing Huang.

**Writing – review & editing:** Xingrui Wang, Xuehong Xiao, Ang Yang, Shuyan Zeng, Wenxi Chen, Yi Chen, Zhihua Huang, Yumei Zeng, Xiaoxing Huang.
